# Impact of Diisocyanates on Morphological and In Vitro Biological Efficacy of Eco-Friendly Castor-Oil-Based Water-Borne Polyurethane Dispersions

**DOI:** 10.3390/polym14173701

**Published:** 2022-09-05

**Authors:** Nadia Akram, Muhammad Usman, Sajjad Haider, Muhammad Saeed Akhtar, Kashmala Gul

**Affiliations:** 1 Department of Chemistry, Government College University Faisalabad, Faisalabad 38000, Pakistan; 2 Chemical Engineering Department, College of Engineering, King Saud University, P.O. Box 800, Riyadh 11421, Saudi Arabia; 3 School of Chemical Engineering, Yeungnam University, Gyeongsan 38541, Korea

**Keywords:** castor oil, IPDI, H_12_MDI, biocompatibility, SEM, biological assay

## Abstract

The search for renewable resources that can replace petroleum products is not only nerve-wracking, but also perplexing, as there is an abundance of plants that have yet to be explored. In this project, virgin castor oil was converted to polyol in two steps: epoxidation and hydroxylation. The resulting polyol was used to synthesize two series of water-borne polyurethane dispersions (WPUDs). The effects of the diisocyanates on the final product were evaluated. Isophorone diisocyanate (IPDI) and dicyclohexylmethane-4,4′-diisocyanate (H_12_MDI) were used as the hard segment (HS) up to 72 wt%, along with 1–4 butanediol (BD) as the chain extender, for the dispersions. Fourier transform infrared spectroscopy (FTIR) confirmed the bonds required for the synthesis of the dispersions. Thermogravimetric analysis (TGA) showed the multistep degradation for both series: maximum degradation took place at 500 °C for IPDI and 600 °C for H_12_MDI-based series. Scanning electron microscopy (SEM) showed phase-segmented morphology. Hemolytic activity was observed at biologically safe levels of up to 7.5% for H_12_MDI-based series. Inhibition of biofilm formation showed comparable results against *Escherichia coli* (*E. coli*) and *Staphylococcus aureus* (*S. aureus*): up to 46%. The results were also confirmed by phase contrast microscopy.

## 1. Introduction

The polymer industry is rapidly devouring petrochemical resources worldwide, which will soon be depleted [1]. There is an urgent need to replace the conventional resources and search for alternative options. So far, it has been found that there is no better alternative than renewable resources for the polymer industry. These renewable resources are relatively inexpensive, environmentally friendly, and harmless [2]. The unabated interest of the global polymer market in reducing pollution has led to the exploration of environmentally friendly polymer products to replace petroleum-based polymers for various applications [3]. Biomass is an abundant renewable material that is different from dwindling petroleum-based materials because its vibrant hydrocarbon components can be converted into raw materials for the polymer industry as monomers. Biomass is a potential candidate to replace the consumption of conventional monomers with environmentally friendly chemicals. Biomass in the form of vegetable oil offers an excellent profile for obtaining biobased compounds [4]. It is an excellent choice for the commercial production of polyol as a feedstock for various polymers, especially polyurethanes (PUs). Currently, PU is used for the production of a wide range of polymer products in almost all fields: transportation, electronics, packaging, coatings and adhesives, etc. PUs are some of the most adaptable polymers, having adjustable properties [5]. The preference for green materials has attracted international attention, as they serve as feedstocks for environmentally friendly polymers. The biomasses of wood, starch, natural fibers, and vegetable oil can be converted into suitable monomers that can be used for the production of PUs [6]. The transition from petroleum-based polymers is not easy because the conventional polyurethane dispersions (PUDs) have been used in countless applications. However, the petroleum-based PUs involve evaporating large amounts of volatile organic compounds (VOCs). These VOCs affect the quality of the air [7]. Environmental pollution is one of the main reasons for replacing these PUs. The removal of VOCs is very costly, and there are no efficient methods that work accurately. Therefore, the only solution is an environmentally friendly alternative to protect the environment [8]. In this context, attempts have been made to replace organic dispersions with WPUDs [9]. WPUDs have proven to be important substitutes for their solvent-based counterparts, as they reduce the harmful health and environmental problems [10].

For the development of any PU, the main components are macrodiol or polyols and isocyanates [11]. Most polyols are derived from petroleum sources, but due to their harmful effects, researchers have turned their interest to environmentally friendly resources such as plant’s oil [6]. The plant oils consist of triglyceride molecules which have ester groups and double bonds, which can be useful for chemical modifications, such as introducing new functional groups [12]. Several effective techniques can be used for the conversion of triglycerides into hydroxyl-containing chains, including: epoxidation, oxirane ring opening, microbial conversion, hydroformylation reduction, and ozonolysis reduction [9]. Castor oil is one of the most important biomasses. It is a pale yellow, viscous liquid derived from castor beans. It consists of 90% fatty acid chains. Castor oil has been used as a source of polyols. The structure of castor oil has an average functionality of 2.7 for hydroxyl groups, which makes it a useful and valuable component for the preparation of PUs in combination with isocyanates and chain extenders [13]. WPUDs have several advantages, including high molecular weight and low viscosity [14]. Plant-oil-based water-borne polyurethane coatings (WPUCs) exhibit antimicrobial properties and are used in biomedical and food packaging applications [15]. These dispersions are nontoxic, nonflammable, environmentally friendly, economically efficient; and offer exceptional chemical resistance, processability, and low temperature flexibility [16,17].

Numerous researchers have contributed to exploring the biomasses that can be used as precursors and finding the influences of different monomers on the final properties of PUs. Athawale and Nimbalkar [18] used sardine fish oil and soybean oil for the preparation of polyurethane dispersions (PUDs) and their inter-esterification products. Cakic et al. [19] used plants oil to prepare PU and silica hybrid dispersions. Nurdin et al. [20] investigated the latest composition of WPUDs using jatropha oil which was functionalized to polyol via the oxirane-ring opening. Mustapa et al. [21] also synthesized PU using jatropha-oil-based polyol. Although there are continuous efforts to explore vibrant renewable resources to produce raw materials for PU, each monomer has a significant impact on the final product. The diversity of PUs lies in the wide variety of potential raw materials. In modern times, significant efforts have been made to find new raw materials and their impacts on properties of PUs. Since the primary architecture of PU consists of hard and soft segments, the choice of monomers plays a crucial role in deciding the PU properties. The novelty of this work is the study of the influences of diisocyanates on the thermal, morphological, and biological properties of castor-oil-derived WPUDs. These dispersions are environmentally friendly and were prepared with the aim of achieving comparable properties to those of PUDs prepared from non-renewable monomers. In this context, the current project has been carried out in two steps. In the first step, the virgin castor oil derived from castor seeds was converted into polyol. In the second step, two cycloaliphatic diisocyanates, IPDI and H_12_MDI, were used to develop two series of WPUDs using BD as chain extenders. The hard and soft segments were systematically varied and analyzed: FTIR, TGA, SEM, and bioassays were performed to compare the influences of diisocyanates.

## 2. Materials and Methods

### 2.1. Materials

The following analytical grade chemicals were used in pure form for the conversion of polyol from castor oil and for the preparation of WPUDs: virgin castor oil derived from castor seeds (Sahiwal market, Pakistan, d = 0.95 g/cm^3^ Mol.wt: 927 g/mol), isophorone diisocyanate (IPDI, d = 1.06 g/cm^3^, (Sigma Aldrich, St. Louis, MO, USA)), dicyclohexylmethane-4,4′-diisocyanate (H_12_MDI, d = 1.05 g/ cm^3^, Sigma Aldrich, St. Louis, MO, USA) anhydrous magnesium sulphate (Sigma Aldrich, St. Louis, MO, USA), 1,4 butanediol (BD, d: 1.02 g/cm^3^ Sigma Aldrich, St. Louis, MO, USA), dimethylol propionic acid (DMPA, (Sigma Aldrich, St. Louis, MO, USA), hydrochloric acid (HCl, Sigma Aldrich, St. Louis, MO, USA), triethyl amine (TEA, Sigma Aldrich, St. Louis, MO, USA), formic acid (reagent grade ≥95%, Merck), hydrogen peroxide (Sigma Aldrich, St. Louis, MO, USA), sulfuric acid (Sigma Aldrich USA), ethyl acetate, (Sigma Aldrich, St. Louis, MO, USA), dibutyl tin dilaurate (DBTDL), Sigma Aldrich, St. Louis, MO, USA)), acetone. Demoisturization of macrodiols was carried out at 70 °C in vacuum for 24 h to remove all impurities and moisture content [3]. Pure bacterial cultures of *Escherichia coli* (*E. coli*) (source: water) and *Staphylococcus aureus* (*S. aureus*) (source: skin infection) were collected from the Department of Microbiology, Government College University, Faisalabad (GCUF). Phosphate buffered saline (PBS; pH~7.4, Sigma Aldrich, St. Louis, MO, USA), triton X-100 (Sigma Aldrich, St. Louis, MO, USA), methanol (Sigma Aldrich, St. Louis, MO, USA), glacial acetic acid (Sigma Aldrich, St. Louis, MO, USA). All chemicals were used without any prior treatment.

### 2.2. Conversion of Castor Oil into Polyol

The virgin castor oil was obtained from castor seeds, and various steps were carried out to obtain the polyol.

#### 2.2.1. Synthesis of Epoxidized Castor Oil

Epoxidized castor oil was prepared by epoxidation of castor oil followed by in situ epoxy ring opening. Epoxidation was performed to convert C=C in castor oil to oxiranes. Castor oil (50 g) was heated to 60 °C in the presence of formic acid (30 g) in a round-bottomed three-neck flask for 40 min. The mixture was acidified by dropwise addition of sulfuric acid (10 g) for 30 min. Epoxidation of the reaction mixture was carried out by adding 40 g of hydrogen peroxide (30 wt%) solution to the acidic oil mixture using a dropping funnel for 2 h at 50 °C. After addition of all reagents, the mixture was gently stirred at 50 °C for seven hours. The mixture was filtered using a separating funnel and rinsed with deionized water until a neutral pH was reached. The oil phase was dried with anhydrous sodium sulphate and then filtered. The moisture contents were removed at 50 °C [22].

#### 2.2.2. Hydroxylation of Epoxidized Castor Oil

The epoxidized castor oil was dispersed in acetone (50 mL) in a round-bottom flask with a magnetic stirrer, in an oil bath at 50 °C. HCl (37%) was added dropwise to the castor oil over a period of 120 min with the constant vigorous stirring. After 2 h of stirring, the acetone was evaporated. The residue was dissolved in a mixture of ethyl acetate and water (3:1). Two layers were separated using a separating funnel. The organic layer was washed with deionized water until a neutral pH was reached. The resulting residue was dried with anhydrous magnesium sulphate. After filtration, the solvent was completely evaporated in a vacuum oven, leaving a viscous yellow product (polyol) [23].

#### 2.2.3. Synthesis of Castor-Oil-Based WPUDs

**Figure 1:**
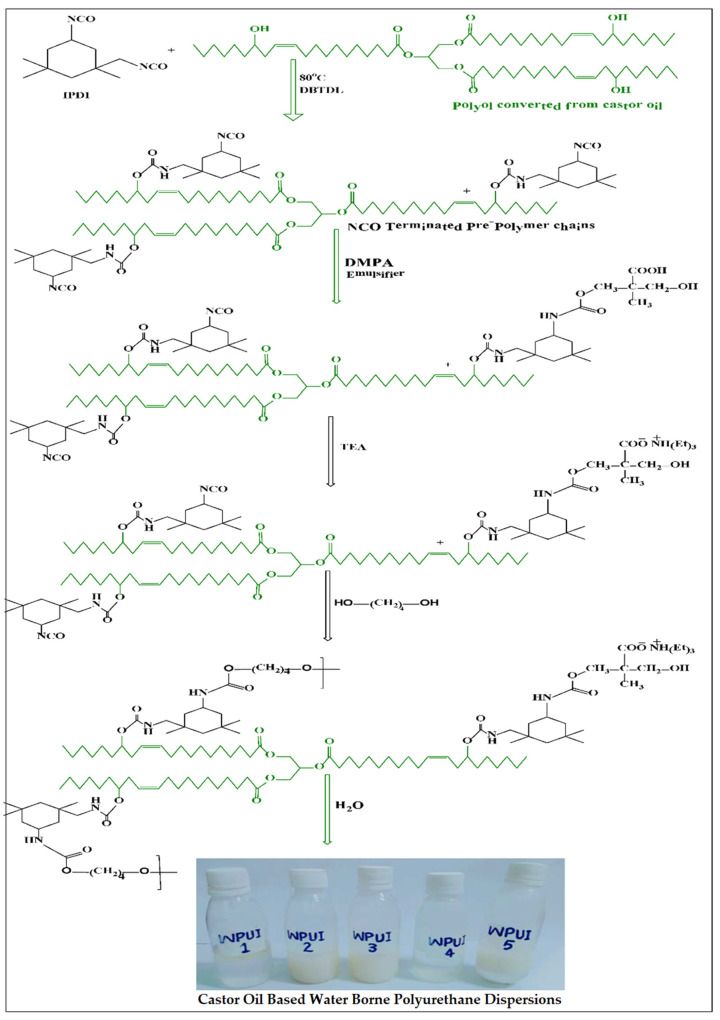
Synthetic route for the bio-derived polyol-based PU dispersions with IPDI for WPUI series representing five stages of dispersion formations, including a first stage of NCO-terminated prepolymer formation, second stage of emulsification with DMPA, third stage of neutralization with TEA, fourth stage of chain extension with BD, and fifth stage of dispersion of polymerized chains in deionized water.

The WPUDs were prepared by in situ polymerization, as described in one of our previous publications [12]. The preparation of the WPUDs was mainly carried out in two steps. The first step was carried out by forming prepolymers, whereas in the second step the prepolymer was converted into polymer chains that were dispersed in deionized water. To carry out this process, the castor-oil-based polyol was treated with stoichiometric amounts of diisocyanates IPDI and H_12_MDI, in two separate series (Table 1). The synthesis was carried out in a four-neck round-bottom flask equipped with a mechanical stirrer, a reflux condenser, a thermometer, and a nitrogen gas supply. The flask was placed on a hot plate in an oil bath. For the preparation of the first series, the weighed amount of castor-oil-based polyol was added to the reaction flask along with IPDI. The reaction was carried out in the presence of DBTDL (1–2 drops) as catalyst and DMPA as internal emulsifier. The chemicals were stirred (at 170–200 rpm) for one hour at 85 °C. The viscosity of the mixture was measured with a digital viscometer and controlled by the addition of acetone. This step resulted in the formation of NCO terminated prepolymer chains. The carboxyl group of DMPA was neutralized by the addition of TEA at 50 °C. The termination of NCO-carrying prepolymers was achieved by the addition of BD. The isocyanate was completely converted according to stoichiometry of the polymers. It is necessary to react all the concentration of isocyanate with polyol in order to avoid byproducts in dispersions. Subsequently, the polymer chains were dispersed in deionized water using a mechanical stirrer at high speed (1300 rpm). A series of five samples was prepared following the same protocol, while systematically varying the molar composition. This series was referred to as the WPUI series. The synthetic pathway is shown in Figure 1. Similarly, a second series of five samples was prepared using castor-oil-based polyol together with H_12_MDI as the diisocyanate. The second series was referred to as the WPUH series. The physical state of the PU dispersion is colloidal. It is a binary colloidal system in which the polyurethane is dispersed in the aqueous dispersion medium.

The hard segment content (HS) of each dispersion was calculated using Equation (1). The HS was developed by the contribution (equivalent weight) of diisocyanate (IPDI or H_12_MDI) along with the chain extender (BD), emulsifier (DMPA), and TEA [24]. The W_total_, on the other hand, indicates the total equivalent weight of all ingredients. The soft segment (SS) was calculated according to Equation (2). The detailed composition of the two series is shown in Table 1. All synthesized samples of the dispersions were collected in polyethylene terephthalate bottles (PET) and stored at room temperature for analysis.

% HS = [(W_IPDI/H12MDI_ + W_DMPA_ + W_TEA_ + W_BD_)/W_total_] × 100 (1)

% SS = 100 − % HS(2)

### 2.3. Characterization

#### 2.3.1. Fourier Transform Infrared Spectroscopy (FTIR)

All monomers and PU dispersions were subjected to FTIR analysis to confirm their functionalities. The spectra were recorded in transmission mode under N_2_ atmosphere at room temperature in the scan range 4000–400 cm^−1^ [25].

#### 2.3.2. Thermal Gravimetric Analysis (TGA)

All synthesized samples were subjected to thermal analysis using Thermo Gravimetric Analyzer (TGA) from Perkin Elmer (Waltham, MA, USA). The thermograms were recorded at a ramp rate of 10 °C min^−1^ under a N_2_ atmosphere ranging from 50 °C to 600 °C. The flow rate of the gas was maintained at 50 mL/min. [2].

#### 2.3.3. Scanning Electron Microscopy (SEM)

Based on HS content (wt%), selected samples of WPUDs were subjected to morphological analysis using a JEOL JSM-7000F coupled with a secondary electron detector (SED). The accelerating voltage was 20 kV, and the spacing was set to 9.0 ± 0.5 mm. Samples with minimum and maximum HS content from both series, WPUI and WPUH, were selected. Before morphological analysis, the dispersions were applied to a cotton cloth and dried at room temperature for three days. The samples were then coated with carbon and the analyses were performed.

#### 2.3.4. Biological Assay

##### Evaluation of Hemolytic Activity

Hemolysis assay was performed with the synthesized WPUDs to evaluate the hemolytic activity. The analysis was performed to investigate the biocompatibility of the synthesized samples. To perform the assay, freshly heparinized human blood (5 mL) was placed in a polystyrene tube. The polystyrene tube was tightly sealed with a screw cap. The polystyrene tube containing the heparinized blood sample was centrifuged at 850 rpm for 5 min. Centrifugation resulted in the separation of supernatant and residue. The supernatant was washed out, and the residue was washed in the form of pellets with phosphate-buffered saline (PBS); pH~7.4. The pellets were washed repeatedly (five times) with PBS solution and then suspended in 20 mL of PBS solution. The suspension containing the pellets was placed in an ice bath. Of this suspension, 180 μL was added to a 2.0 mL microcentrifuge tube. A small amount of WPUD (20 μL) was also added to a microcentrifuge tube and mixed homogeneously. PBS was used as the negative control of the experiment, and Triton X-100 (0.1%) served as the positive control. The microtubes were immediately centrifuged for 10 min. The resulting supernatant (100 μL) was collected and diluted with PBS (900 μL). Three replicates of the supernatant (200 μL each) were placed in a sterile microplate containing a positive and a negative control. Absorbance was measured at 540 nm using a microplate reader (BioTek, Winooski, VT, USA). Hemolysis (%) of each sample was calculated according to Equation (3) [26,27].

(3) $$\;\mathrm{Hemolysis}\;\left(\%\right)=\frac{\mathrm{Sample}\;\mathrm{absobance}-\mathrm{negative}\;\mathrm{control}\;}{\mathrm{Positive}\;\mathrm{control}}\times 100$$

##### Biofilm Inhibition Assay

The development of a bacterial biofilm depends on the growth of bacterial cells and their interactions with the surrounding medium. This assay was performed to evaluate the potential of WPUDs to inhibit the development of a bacterial film. The ability to inhibit biofilm formation was investigated against *S. aureus* and *E. coli* [28,29]. The experiment was performed using a sterilized plastic tissue culture in a 96-well plate with a plain bottom filled with 100 μL of nutrient broth (Oxoid, Basingstoke, UK). In total, 100 μL of WPUD was inoculated into the nutrient broth. The bacterial culture and its nutrient broth served as controls for both strains (10 μg/20 μL) without WPUD sample. To compare WPUD results, ciprofloxacin was used as a standard (positive control). The 96-well plates were incubated under aerobic conditions at 37 °C for 24 h. After incubation, each well was washed three times with sterile PBS. Before washing, adherent bacteria were washed with 99% methanol (220 μL per well), and the mixture was allowed to settle for 15 min. The excess methanol was removed. Staining was performed with 2% crystal violet dye (220 μL), which was added to each well and left for 5 min. The excess dye was washed off with water and air dried at room temperature. The dye adhering to the adherent cells was redissolved with glacial acetic acid (33% (*v*/*v*)). The microplate reader (BioTek, Winooski, VT, USA) was used to measure the optical density (OD) in each well at 630 nm. Equation (4) was used to calculate the percentage of bacterial growth inhibition (*INH*%).

(4) $$INH\left(\%\right)=1-\frac{\mathrm{OD}630\;\left(\mathrm{sample}\right)}{\mathrm{OD}630\;\left(\mathrm{control}\right)}\times 100\%$$

##### Phase Contrast Microscopic Analysis

Phase contrast microscopy was used to confirm the analysis of biofilm inhibition of polyurethane dispersions [30]. The analysis was performed with a few drops of *S. aureus* and *E. coli* cultured on glass slides. Incubation was performed at 37 °C for 14 h. The slides were rinsed with PBS solution, and after washing the slides, WPUDs were immersed on them. The slides were washed and stained, and the biofilms were dissolved with glacial acetic acid (30%). Negative PBS control and positive control slides with ciprofloxacin were also prepared without PU samples. All slides prepared during analysis were examined under the microscope.

## 3. Results and Discussion

### 3.1. Identification of Functional Groups and Linkages by FTIR

**Figure 2:**
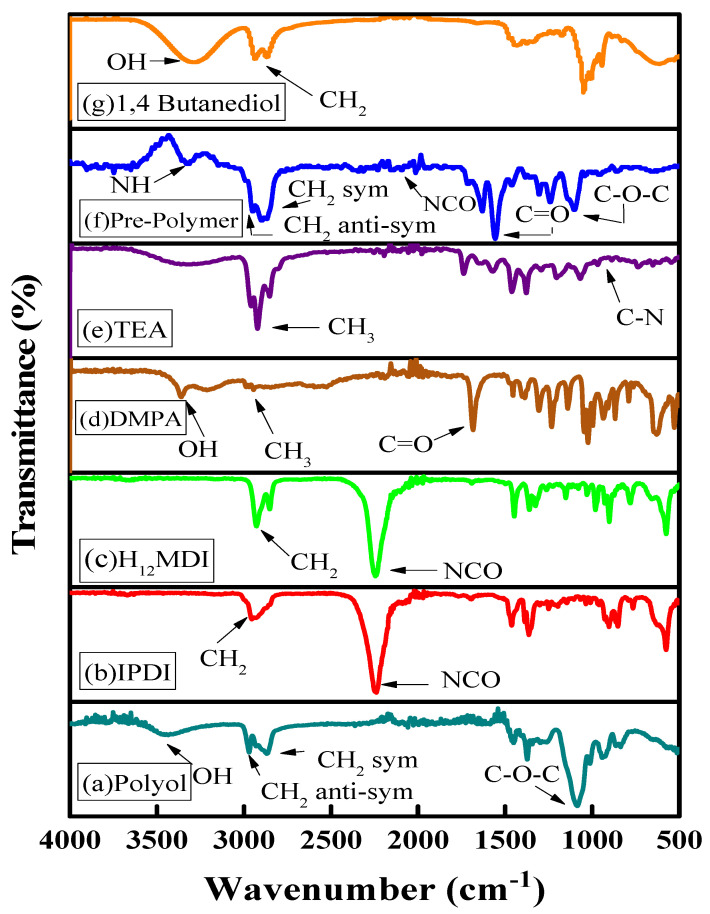
FTIR spectra of monomers and the prepolymer of WPUDs dispersions. (**a**) Polyol: the spectrum of the castor-oil-derived monomer; (**b**) IPDI: the spectrum of isophorone diisocyanate; (**c**) H_12_MDI: the spectrum of dicyclohexylmethane-4,4′-diisocyanate; (**d**) DMPA: the spectrum of dimethylolpropionic acid; (**e**) TEA: the spectrum of triethylamine; (**f**) the spectrum of prepolymer; (**g**) the spectrum of the chain extender.

The FTIR spectra of all monomers and the different steps of water-borne PU dispersion are shown in Figure 2. The monomers showed their specific bands, such as the polyol derived from castor oil, showed the -OH group bending band at 3389 cm^−1^. The bands at 2926, 2855, and 1739 cm^−1^ were designated to asymmetric stretching and symmetric stretching of the CH_2_ group and C=O group, respectively. The NCO group of isocyanates present in IPDI and H_12_MDI showed its characteristic band at 2246 cm^−1^. A broad band at 3653 cm^−1^ was observed in the spectrum of DMPA, indicating the existence of the -OH group. The FTIR spectrum of BD showed a broad band at 3277 cm^−1^, which also indicated the presence of the -OH group. The FTIR spectra of NCO terminated pre-polymer showed the presence of the -NH group at 3473 cm^−1^, along with the stretching bands of the CH_2_ group and carbonyl group at 2959 and 1739 cm^−1^, respectively. The stretching band of the -CH group was observed in the range of 2700 to 2950 cm^−1^; the stretching band of the -NH group was observed in the range of 3500 to 3300 cm^−1^. No band indicating the presence of NCO in the spectra of the PU dispersions was observed, suggesting that all NCO groups are consumed in the urethane bond development without urea being formed as a by-product. A broad band in the region of 3200 cm^−1^ was attributed to water, which was used as a solvent. The presence of all expected functional groups and bands at their respective wavenumbers indicated the successful completion of reaction at each step, and WPUDs’ synthesis. The FTIR results documented by our group and other authors for the synthesis of water-based PUs are in agreement [31,32].

### 3.2. Thermal Stability of WPUDs Using Thermogravimetric Analysis

**Figure 3:**
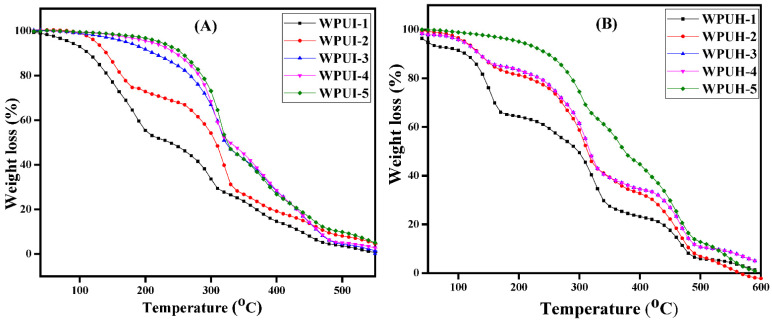
Thermogravimetric analysis of WPUDs: (**A**) WPUI series and (**B**) WPUH series.

The TGA curves of the WPUI and WPUH series are shown in Figure 3A,B. Multi-step curves were observed for both series. The data of onset temperature, endset temperature along with sample residues (%) for both series have been shown in Table 2. The minimum onset temperature was measured to be 90 °C for the WPUI series and 80 °C for the WPUH series. The end set temperature was 500 °C or above. For WPUI-5 and WPUH-5 with 72 (wt%) HS, the thermal stability was observed to remain at 230 and 220 °C, respectively. It was observed that the gradual increase in HS from 59 to 72 wt% increased the onset temperature from 90 to 230 °C for WPUI series and from 80 to 220 °C for WPUH series. Three-step degradation was observed in both series. The first step was the dissociation of PU into monomers (isocyanate and alcohol). The dissociation occurred very slowly in the temperature range between 150 and 200 °C. The second step consisted of the degradation of the HS residues to propane and carbon dioxide. This happened in a temperature range of 250 to 350 °C. The last step was the decomposition of the hydrocarbon chains of SS (polyol). This decomposition occurred above 500 °C. The thermal stability of water-borne PU mainly depends on the thermal stability of HS. A similar trend has been observed for both WPUI and WPUH series, but the thermal stability and degradation temperature were slightly lower for the WPUH series. Our results are in agreement with previously published results. [33].

### 3.3. Morphological Characterization

**Figure 4:**
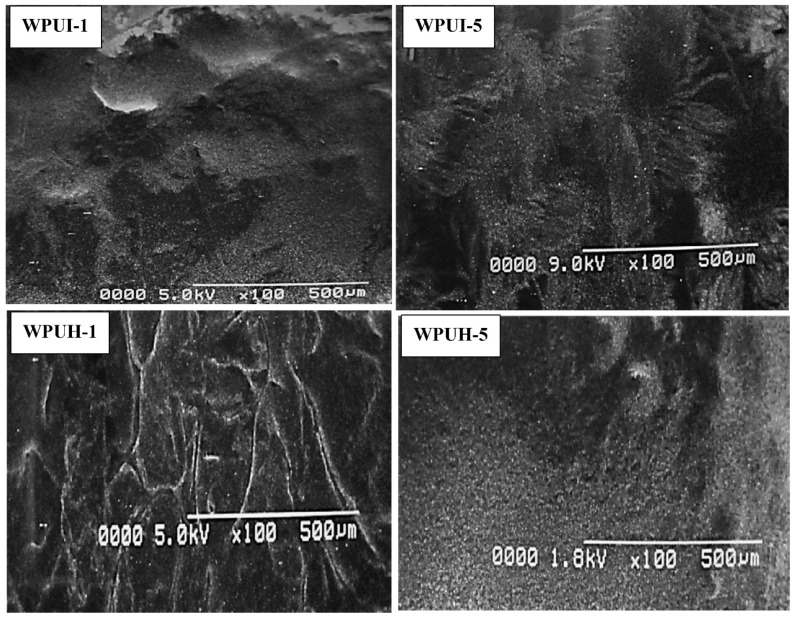
SEM micrographs of WPUI-1, WPUI-5, WPUH-1, and WPUH-5. The dark areas represent the soft segments, and the bright areas represent the hard segments. All images show a magnification of 100× with a resolution of 500 μm.

Morphology is important to illustrate the homogeneity of a structure. PUs are multipurpose polymers, not only because of their versatility in raw materials, but also because of their significant segmentation of HS and SS. Various factors, such as chemical structure resulting from the different components, the nature of SS, the stiffness of the chains, and the viscosity, play important roles in determining the morphology of PU. The microscopic images of the samples show a clear distinction of segmentation. There are evenly distributed bright and dark areas indicating interactions between the two phases. The bright areas represent HS, of diisocyanate and chain extender, and the dark areas represent SS, of polyol (Figure 4). The bright areas increased in WPUI-5 due to the larger contribution of HS. Similar results were also observed in WPUH-5. Moreover, the increases in HS (%) in WPUI-5 and WPUH-5 showed slight increase in the roughness compared to WPUI-1 and WPUH-1, indicating that stoichiometry must be controlled to achieve optimal performance of the polymers [34]. Researchers have reported such structures of PUs prepared with cardanol-diol using SEM. Reignier et al. [35] have also reported the segmented phase morphology of PUs using SEM. Allauddin et al. [36], however, reported microscopic images without phase separation for castor-oil-based polyurethane-urea coatings when the chlorine content in the coatings was increased. This supports our argument that the properties of PU dispersions vary with composition and stoichiometry. The morphological results of our study are consistent with those of previously reported studies [35,36].

### 3.4. Evaluation of In Vitro Biological Efficacy/Biocompatibility WPU Dispersions

#### 3.4.1. Hemolytic Activity of WPU Dispersions

**Figure 5:**
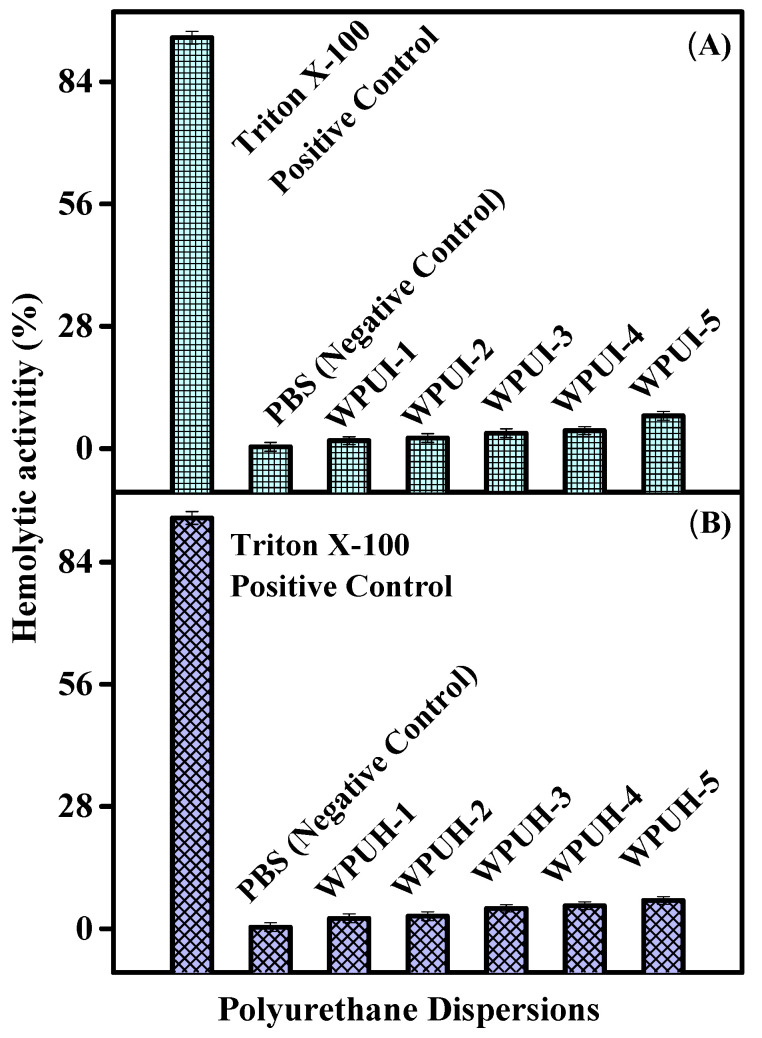
Hemolytic activity of polyurethane dispersions. (**A**) Hemolytic activity of WPUI. (**B**): Hemolytic activity of WPUH series.

The in vitro biological analysis data are shown in Table 3, which summarizes the hemolytic activity of the WPUI and WPUH series. The graphical representations of the hemolytic activity of the two series are shown in Figure 5. Biologically, hemolysis is a process in which erythrocytes (red blood cells; RBCs) burst or rupture. In this process, the blood cells are completely ruptured and the entire contents are removed. The synthesized WPUDs are intended to be inherently biocompatible. Further, the tendency of PU dispersions to damage the membrane is based on the release of hemoglobin. Therefore, a hemolytic assay was performed to evaluate the biocompatibility of the synthetic WPUDs, which provided information about the hemolysis of the dispersions. The positive control in the assay was Triton-X 100, which had a hemolysis value of 94.1 ± 0.28%, making it very harmful to erythrocytes. In contrast, the negative control for the assay, phosphate buffer saline (PBS), had a maximum hemolysis value of 0.41 ± 0.10%, indicating a minimal tendency for erythrocyte rupture. Both the upper (Triton-X 100) and lower limits (phosphate buffer) were used as standard references in this study. In the WPUI series, the minimum value of hemolytic activity was 2.4 ± 1.00%, and the maximum value of hemolytic activity was 6.5 ± 0.91%. The results show that all samples had very low hemolytic activity compared to the positive control, and therefore, the products are biocompatible and safe for biomedical applications. The results are also in agreement with the findings of Misbah et al. [5], who found hemolytic activity of 10.59% for sodium-alginate-based polyurethane dispersions when using IPDI as diisocyanate. Similarly, WPUH series showed the minimum hemolytic value of 1.8 ± 0.91% for WPUH-1, and the maximum hemolytic value of 7.5 ± 11% was much lower than that of the standard value of positive control. The results successfully demonstrate the biocompatibility of the synthesized samples.

#### 3.4.2. Biofilm Inhibition of Polyurethane Dispersions

**Figure 6:**
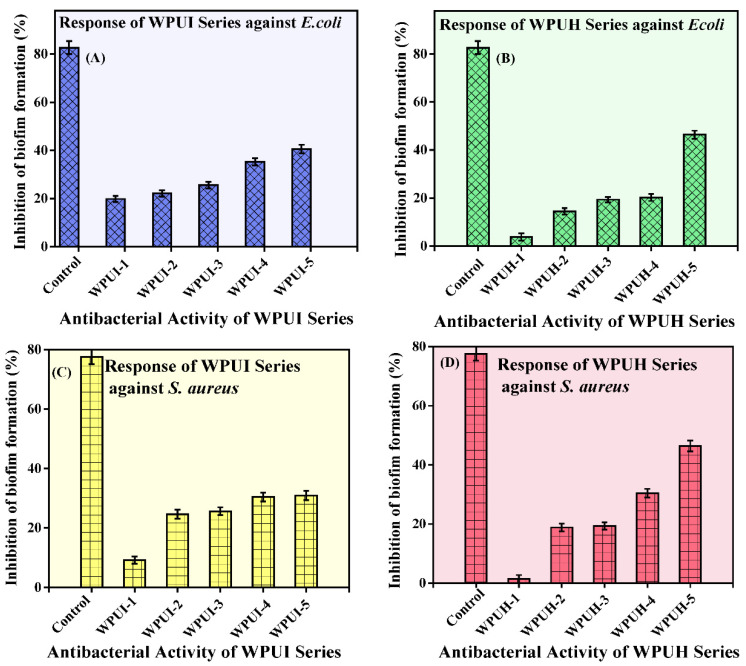
Biofilm inhibitory activity of the WPUDs. (**A**) WPUI series against *E. coli*. (**B**) WPUH series against *E. coli*. (**C**) WPUI series against *S. aureus*. (**D**) WPUH series against *S. aureus*.

The ability of the two WPUDs series to inhibit bacterial biofilm formation was evaluated against two bacterial strains: *E. coli* and *S. aureus*. The data are presented in Table 3 and Figure 6. Ciprofloxacin, an antibacterial drug, was used as a control against *E. coli* and *S. aureus*. Ciprofloxacin showed biofilm inhibitory activity of 77.57 ± 2.37% against *S. aureus* and 82.65 ± 2.74% against *E. coli*. The WPUI series samples showed biofilm inhibitory activity ranging from 19.81 ± 1.25 to 40.58 ± 1.8 against E. coli and 9.2 ± 1.21% to 30.9 ± 1.61% against *S. aureus*. WPUH series, on the other hand, showed better biofilm inhibitory activity ranging from 3.86 ± 1.50% to 46.38 ± 1.71% against *E. coli* and 1.4 ± 0.41 to 46.4 ± 1.80% against *S. aureus*. Moreover, the biofilm inhibitory activity of the WPUDs against both bacteria increased with an increase in the content of HS. When comparing the effect of diisocyanate, the H_12_MDI (WPUH)-containing samples showed better biofilm inhibitory activity than the IPDI (WPUI)-containing samples. However, the difference was small, indicating that any diisocyanate can be used effectively. The small difference caused by any monomer can be compensated for by stoichiometric adjustments. In general, the WPUI series samples showed better resistance to *E. coli*, whereas the WPUH series samples showed comparable resistance to both *E. coli* and *S. aureus* bacterial strains. These analyses are good evidence that structural modifications alone can significantly improve the biocompatibility of WPUDs and that the synthesized environmentally friendly dispersions are appropriate to being used in biomedical applications.

#### 3.4.3. Phase Contrast Microscopic Analysis of Polyurethane Dispersions

**Figure 7:**
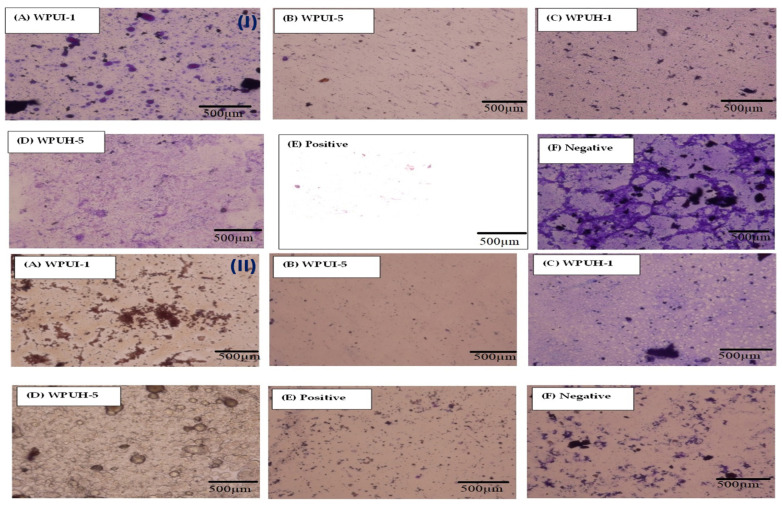
Phase contrast microscopy showing the pattern of bacterial biofilm formation and its inhibition (**I**). (**A**,**B**) WPUI-treated slides. (**C**,**D**) WPUH-treated slides. (**E**) Positive growth and (**F**) negative growth against *E. coli* biofilm. (**II**) (**A**,**B**) WPUI-treated slides. (**C**,**D**) WPUH-treated slides. (**E**) Positive growth and (**F**) negative growth against *S. aureus* biofilm.

Phase contrast microscopy was used to visually examine the formation of biofilms by bacterial strains *E. coli* and *S. aureus* and the tendency of the WPUDs to inhibit bacterial growth. In Figure 7, the micrographs show massive growth of *E. coli* in the case of the negative control (sample without antibacterial component or WPUDs (Figure 7I(F)), and there was little evidence of bacterial growth in the case of the positive control (ciprofloxacin, Figure 7I(E)). The two control samples were used as standards to study the inhibition of biofilm formation. The micrographs of the selected samples of WPUI (Figure 7I(A,B)) and WPUH (Figure 7I(C,D)) against *E. coli* are shown in Figure 7. WPUI-1 (Figure 7I(A)) showed a micrograph very similar to the negative control, indicating that the sample had lower efficacy against *E. coli* biofilm formation, and the micrograph of WUPI-5 (Figure 7I(B)) is very similar to that of the positive control, indicating that the sample had better resistance against *E. coli* biofilm formation. A similar effect was also observed for WPUH-1 and WPUH-5, indicating that the samples showed better defense against *E. coli* biofilm formation with higher concentrations of HS. Similar to the study with *E. coli*, *S. aureus* grew in the negative control (Figure 7II(F)), but there was little evidence of bacterial growth in the positive control ((Figure 7II(E)). In addition, low efficacy of WPUI-I (Figure 7II(A)) and WPUH-I (Figure 7II(C)) against *S. aureus* biofilm formation was observed. However, WPUI-5 (Figure 7II(B)) and WPUH-5 (Figure 7II(D)) showed better defense.

From the results, it can be concluded that the series showed similar trends against the selected bacterial strains, as shown by the biofilm inhibition of the polyurethane dispersions (Section 3.4.2). The data also confirm the suggestion that the samples inhibit microbial biofilm growth. The results indicate that the WPUDs have good biocompatibility and can be considered for appropriate biomedical applications.

## 4. Conclusions

The rapid consumption of petroleum-based polymers and the associated pollution have prompted researchers to search for alternative raw materials, preferably from renewable resources. The environmentally friendly products have the ability to protect the environment and health alike. In the current project, castor-oil-based polyol was used to produce biocompatible water-borne PU dispersions. Two different series of WPUD were synthesized using IPDI and H_12_MDI. FTIR analysis confirmed the presence of all the characteristic functionalities necessary for the development of PUs. Thermal stability was observed at up to 500 °C by TGA. The samples showed segmented morphology due to the contributions of HS and SS. The biocompatibility of the synthesized samples was evaluated by measuring the hemolytic activity. WPUI series showed better results (6.5 ± 0.91) compared to WPUH (7.5 ± 1.0) at an optimal concentration of HS (72%). Percent inhibition of biofilm formation was evaluated against E. coli and *S. aureus*. WPUH series samples showed better resistance against both bacterial strains, 46.38 ± 1.71% and 46.4 ± 1.80%, compared to WPUI-based samples. The results were also verified by phase contrast microscopy. Both diisocyanates, H_12_MDI and IPDI, are equally capable of producing environmentally friendly and biocompatible polyurethane dispersions. This makes the castor-oil-derived polyurethane dispersions potential candidates for biomedical applications.

## Figures and Tables

**Table 1:** Sample code designation, stoichiometry, and physical properties of WPUD.

**Series—I (WPUI)**	**Molar Composition** **(mol.%)**				**Segmentation in PU Dispersions (%)**	
**Sample Code**	**Polyol**	**DMPA**	**IPDI**	**BDO**	**Soft Segment** **(SS)**	**Hard Segment** **(HS)**
WPUI-1	1	1	3.5	1.0	41.0	59.0
WPUI-2	1	1	4.0	1.5	35.0	65.0
WPUI-3	1	1	5.0	2.5	32.2	67.8
WPUI-4	1	1	6.0	3.5	29.2	70.8
WPUI-5	1	1	7.0	4.5	28.0	72.0
**Series—II (WPUH)**	**Molar Composition** **(mol.%)**				**Segmentation in PU dispersions (%)**	
**Sample Code**	**Polyol**	**DMPA**	**H_12_MDI**	**BDO**	**Soft Segment** **(SS)**	**Hard Segment (HS)**
WPUH-1	1	1	3.5	1.0	47.7	52.3
WPUH-2	1	1	4.0	1.5	42.7	57.3
WPUH-3	1	1	5.0	2.5	38.5	61.5
WPUH-4	1	1	6.0	1.0	35.2	64.8
WPUH-5	1	1	7.0	4.5	32.2	67.8

WPUI represents the WPUDs series with IPDI as diisocyanate, and WPUH represents the WPUDs series with H_12_MDI as diisocyanate (the digits shown with WPUI and WPUH from 1 to 5 represent the systematic variation of HS).

**Table 2:** Thermal analysis of WPUI and WPUH series.

Sample Code	T_on set_ °C	T_end set_ °C	Sample Residue (%)	Sample Code	T_on set_ °C	T_end set_ °C	Sample Residue (%)
WPUI-1	90	580	0.54	WPUH-1	80	590	0.87
WPUI-2	99	580	0.29	WPUH-2	110	560	0.57
WPUI-3	160	570	3.24	WPUH-3	112	590	5.02
WPUI-4	210	600	0.08	WPUH-4	119	590	5.02
WPUI-5	230	580	2.97	WPUH-5	220	590	1.32

**Table 3:** Data of bioassays of WPUDs of WPUI and WPUH series.

Sample Code	Hemolytic Activity (%)	Inhibition of Biofilm Formation Against		Sample Code	Hemolytic Activity (%)	Inhibition of Biofilm Formation Against	
		*E. coli*	*S. aureus*			*E. coli*	*S. aureus*
WPUI-1	2.40 ± 1.00	19.81 ± 1.25	9.2 ± 1.21	WPUH-1	1.80 ± 0.91	3.86 ± 1.50	1.4 ± 0.41
WPUI-2	2.90 ± 1.00	22.10 ± 1.30	24.6 ± 1.52	WPUH-2	2.40 ± 1.00	14.49 ± 1.31	18.8 ± 1.30
WPUI-3	4.70 ± 0.80	25.6 ± 1.40	25.6 ± 1.31	WPUH-3	3.50 ± 1.00	19.32 ± 1.21	19.3 ± 1.21
WPUI-4	5.30 ± 0.91	35.27 ± 1.50	30.4 ± 1.50	WPUH-4	4.10 ± 0.91	20.29 ± 1.50	30.4 ± 1.50
WPUI-5	6.50 ± 0.91	40.58 ± 1.8	30.9 ± 1.61	WPUH-5	7.50 ± 1.00	46.38 ± 1.71	46.4 ± 1.80
Triton X-100 (Positive Control)	94.10 ± 0.28	-	-	Triton X-100 (Positive Control)	94.10 ± 0.28	-	-
PBS (Negative control)	0.41 ± 0.10	-	-	PBS (Negative control)	0.41 ± 0.10	-	-
Ciprofloxacin (positive control)	-	82.65 ± 2.74	77.57 ± 2.37	Ciprofloxacin (positive control)	-	82.65 ± 2.74	77.57 ± 2.37

## Data Availability

The data presented in this study are available on request from the corresponding author.
